# Modified Nucleotides as Substrates of Terminal Deoxynucleotidyl Transferase

**DOI:** 10.3390/molecules22040672

**Published:** 2017-04-22

**Authors:** Daiva Tauraitė, Jevgenija Jakubovska, Julija Dabužinskaitė, Maksim Bratchikov, Rolandas Meškys

**Affiliations:** 1 Department of Molecular Microbiology and Biotechnology, Institute of Biochemistry, Life Sciences Center, Vilnius University, Sauletekio al. 7, Vilnius LT-10257, Lithuania; jevgenija.jakubovska@bchi.vu.lt (J.J.); julija.dab@gmail.com (J.D.); rolandas.meskys@bchi.vu.lt (R.M.); 2 Department of Physiology, Biochemistry, Microbiology and Laboratory Medicine, Faculty of Medicine, Vilnius University, M. K. Čiurlionio g. 21, Vilnius LT-03101, Lithuania; maksim@pcr.lt

**Keywords:** terminal deoxynucleotidyl transferase, *N*^4^-amino-2’-deoxycytidine triphosphate, 4-thio-2’-deoxyuridine triphosphate, 4-bromo-2-pyridone, 4-chloro-2-pyridone, UV cross-linking

## Abstract

The synthesis of novel modified nucleotides and their incorporation into DNA sequences opens many possibilities to change the chemical properties of oligonucleotides (ONs), and, therefore, broaden the field of practical applications of modified DNA. The chemical synthesis of nucleotide derivatives, including ones bearing thio-, hydrazino-, cyano- and carboxy groups as well as 2-pyridone nucleobase-containing nucleotides was carried out. The prepared compounds were tested as substrates of terminal deoxynucleotidyl transferase (TdT). The nucleotides containing *N*^4^-aminocytosine, 4-thiouracil as well as 2-pyridone, 4-chloro- and 4-bromo-2-pyridone as a nucleobase were accepted by TdT, thus allowing enzymatic synthesis of 3’-terminally modified ONs. The successful UV-induced cross-linking of 4-thiouracil-containing ONs to TdT was carried out. Enzymatic post-synthetic 3’-modification of ONs with various photo- and chemically-reactive groups opens novel possibilities for future applications, especially in analysis of the mechanisms of polymerases and the development of photo-labels, sensors, and self-assembling structures.

## 1. Introduction

Oligonucleotides (ONs) bearing nucleobase or carbohydrate backbone modifications have a wide range of applications such as the development of aptamers [1,2], specific labelling of nucleic acids or proteins [3], functionalization of surfaces [4], scaffolding of biocatalysts [5] or creation of biosensors [6]. Different strategies are employed to insert functional groups into ONs. The most common are synthesis by the standard solid-phase phosphoramidite chemistry [7], enzymatic synthesis by DNA or RNA polymerases using modified (2’-deoxy)ribonucleoside 5’-triphosphates (dNTPs or NTPs) as substrates [8,9,10,11], post-synthetic modification [12,13] and DNA ligation [14]. A broad spectrum of modified dNTPs as well as diverse polymerases have been tested and utilized for the synthesis of sugar-, phosphate- or base-modified DNA [15,16,17,18,19]. A straightforward approach to enzymatically prepared modified ONs is DNA end labelling based on terminal deoxynucleotidyl transferase (TdT) [20,21]. TdT adds random nucleotides at the 3’-OH terminus of DNA using dNTPs as substrates in a completely template-independent manner. Beyond its natural substrate, TdT is known to use a wide variety of nucleoside triphosphate analogues such as *p*-nitrophenyl triphosphate [22], 5-substituted indolyl deoxynucleotides [23], dinucleoside 5’,5’-tetraphosphate [24], pyrene-nucleoside triphosphate [25,26] or benzo-expanded dNTP [27]. Several recent reports demonstrate a modified nucleotide incorporation by TdT in order to provide nuclease resistance [28], introduce a functional group [29] or upgrade 3’-end labelling with “click” chemistry [30]. The crystal structure of TdT [31] provides a better understanding of reaction mechanism, however details regarding nucleotide selection remains elusive.

Halogenated nucleotides such as 5-bromo-2′-deoxyuridine (BrdU) and 5-iodo-2’-deoxyuridine (IdU) have been comprehensively studied as photo-reactive chromophores [32,33,34,35]. Toxic effects caused by UVB and UVC radiation are enhanced when BrdU or IdU is present in DNA, while these are UVA photosensitizers only along with DNA-intercalating UVA chromophore [36]. An alternative strategy to photoactivate DNA by UVA is to use modified nucleotides which may shift absorbance maximum to UVA region [37]. In addition, modification of nucleobase might improve radiosensitizing properties under hypoxic conditions as in the case of 4-bromo- and 5-bromopyridone analogues of BrdU [38]. Despite above mentioned properties, halogen-containing ONs may be further modified during palladium-catalysed cross-coupling [39]. Altogether, halogenated nucleotides and ONs not only contribute to investigation of DNA lesions but also act as precursors for post-synthetic modifications.

Thio group-bearing (deoxy)ribonucleotides have been extensively used as intrinsic photolabels to analyse the three-dimensional structure of RNA molecules, to identify contacts between nucleic acids and proteins, to detect epitranscriptomic marks, and to manipulate individual DNA molecules [40,41,42,43,44]. Similarly to halogenated nucleotides, thio-nucleotides such as 4-thio-2’-deoxyuridine (4-thiodU), 4-thio-5-bromo-2’-deoxyuridine (4-thio-5-Br-dU), 6-thioguanosine (6-thioG) or 4-thiothymidine (4-thioT) operate as UVA sensitizers while the latter is easily incorporated into DNA during replication [45,46,47].

Recently, much attention has been paid to the preparation of hydrazine functionality-containing nucleotides and ONs [48,49,50,51,52,53]. It has been shown that *N*^4^-aminocytidine induces an AT to GC transition and can function as a mutagen [54]. Compared to well-known amino-modified ONs, hydrazines exhibit enhanced reactivity with active esters at neutral to acidic conditions as well as generate more stable adducts—hydrazones—reacting with aldehydes [50]. Usually, the hydrazine group is introduced into ON post-synthetically [48,49,52,53] and only recently the protected hydrazine amidites have been reported [50,51]. Both conjugation and immobilization of ONs continue to develop towards high-throughput technologies, therefore significance of hydrazine-modification is obvious. Pyridone-, cyano- and carboxy-functionalities are also worth mentioning since they open many ways for further chemical or enzymatic modifications of nucleobases [55,56,57,58].

Whereas the utility of thio- or hydrazine-modified ONs is evident, the lack of convenient building blocks as well as methods for their introduction into ONs has thus far hindered the wide use of these functional modifications. Here we present a synthetic approach to produce triphosphates of 4-thio-2’-deoxyuridine and 2’-deoxycytidine analogue bearing hydrazine group at the 4th position. To the best of our knowledge, the template-independent enzymatic labelling approach has not yet been used for the synthesis of 3’-modified ONs with 4-thio-2’-deoxyuridine, *N*^4^-amino-2’-deoxycytidine or 4-Br(Cl)-pyridones.

## 2. Results and Discussion

### 2.1. Synthesis of Modified Nucleotides

**Scheme 1:**
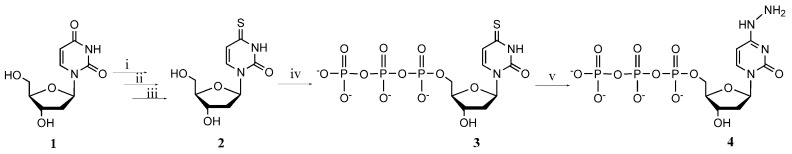
Synthesis of 4-thio-2’-deoxyuridine triphosphate (**3**) and *N*^4^-amino-2’-deoxycytidine triphosphate (**4**). *Reagents and conditions*: (i) Ac_2_O, NaOAc, 90 °C, 30 min; (ii) Lawesson’s reagent, toluene, 90 °C, 2 h; (iii) NaOCH_3_, CH_3_OH, rt, 30 min; (iv) POCl_3_, Bu_3_N, trimethyl phosphate, 0 °C, 90 min; then Bu_3_N, (NHBu_3_)_2_H_2_P_2_O_7_, CH_3_CN, 0 °C, 15 min; (v) NH_2_-NH_2_, H_2_O, rt, 18 h.

4-Thio-2’-deoxyuridine (**2)** was prepared by adapting and modifying methods reported in the literature [59,60]. A general synthetic approach consists of three stages: (i) protecting the hydroxyl groups on the sugar by acylation; (ii) replacing the oxygen atom at 4-position with a sulphur atom using Lawesson‘s reagent; and (iii) removing the protecting groups with sodium methylate to produce the 4-thio-2’-deoxyuridine. The overall yield of three steps was 63%. Synthesized 4-thio-2’-deoxyuridine was converted to the nucleoside triphosphate according to the one-pot phosphorylation method. *N*^4^-Amino-modified cytidine triphosphate could be prepared via bisulphite-mediated displacement of the C4 amino group of cytosine, for example *N*^4^-amino-2’-deoxycytidine triphosphate was synthesized from 2’-deoxycytidine triphosphate (dCTP) via microwave-mediated bisulphate-catalysed transamination with hydrazine under strict control of reaction conditions [61]. In this research we synthesized *N*^4^-modified dCTP directly and simply from the prepared 4-thio-2’-deoxyuridine triphosphate in 66% yield at room temperature (Scheme 1).

**Scheme 2:**
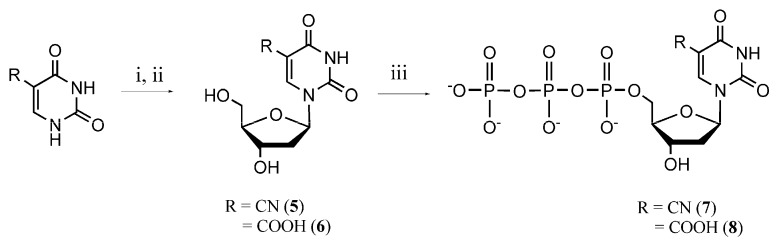
Synthesis of 5-cyano- and 5-carboxy-dUTP. *Reagents and conditions*: (i) 1,1,1,3,3,3-hexamethyldisilazane (HMDS), trimethylsilyl chloride (TMSCl), 80 °C, 4h; then 1,3,5-*O*-triacetyl-2-deoxyribose, SnCl_4_, rt, 5 h; (ii) NaOCH_3_, CH_3_OH, rt, 30 min; (iii) POCl_3_, Bu_3_N, trimethyl phosphate, 0–4 °C, 2–3 h; then Bu_3_N, (NHBu_3_)_2_H_2_P_2_O_7_, CH_3_CN, 0 °C, 10–15 min.

5-Cyano-2’-deoxyuridine can be synthesized by treatment of 2’-deoxy-5-iodouridine with KCN, or by treatment of 5-trifluoromethyl-2’-deoxyuridine with concentrated NH_4_OH [62]. 5-Carboxy-2’-deoxyuridine can be obtained from the oxidation of thymidine by menadione-mediated photosensitization [63] or from the alkaline hydrolysis of 5-trifluoromethyl-2’-deoxyuridine [64]. In this work 5-substituted 2’-deoxyuridine derivatives were obtained via glycosylation of silylated 5-cyano- or 5-carboxy-uracil with acetylated 2-deoxyribose in the presence of tin (IV) chloride as Lewis acid catalyst. Synthesized 5-substituted nucleosides were converted to the nucleotides according to the one-step phosphorylation method (Scheme 2).

This method allowed accessing the triphosphate from the appropriate modified nucleoside in 20% yield. The triphosphates were best isolated by ion exchange chromatography using a diethylaminoethyl (DEAE) Sephadex A-25 column with a gradient of LiCl, followed by precipitation of modified nucleotides from acetone/methanol mixtures. The one step phosphorylation procedure allowed us to prepare the nucleoside triphosphates with >95% purity in a fast way for further studies.

### 2.2. Incorporation of Modified Nucleotides by Terminal Deoxynucleotidyl Transferase

In order to determine whether these novel modified nucleotides can be incorporated into ON, primer extension reactions (PEX) were conducted using TdT. Among all DNA polymerases TdT is unique for its broad utilization of divalent metal ions. TdT is able to use several metal cations such as Mg^2+^, Zn^2+^, Co^2+^, Mn^2+^ [65]. Furthermore, each divalent metal ion contributes to the kinetics of nucleotide incorporation differently. It has been shown that in the presence of Co^2+^ TdT preferentially incorporates purine nucleotides while Mg^2+^ facilitates incorporation of pyrimidine nucleotides [66]. Zn^2+^ can also improve template-independent synthesis [67]. To monitor the discrimination between incorporation of modified nucleotides by TdT two buffer systems were used. In our studies the first system is based on sodium glutamate and Mg^2+^ which is likely to be the basic enzyme cofactor in vivo. Contrary, another buffer system which is referred to as optimal buffer for TdT is absolutely artificial compared to in vivo conditions as it contains potassium cacodylate and Co^2+^. Moreover thinking of Co^2+^ as a versatile metal ion cofactor capable of playing a role of both Mg^2+^ and Zn^2+^, a more efficient nucleotide incorporation using latter buffer system could be anticipated [68].

**Figure 1:**
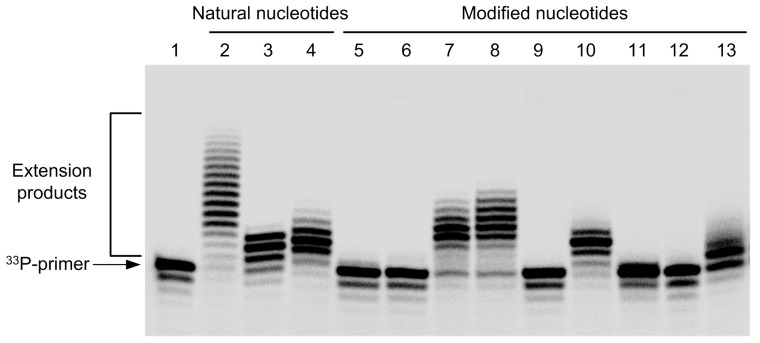
Polyacrylamide gel electrophoresis (PAGE) analysis of primer extension reactions (PEX) with terminal deoxynucleotidyl transferase (TdT) in glutamate/Mg^2+^ buffer. Lane 1, primer labelled at the 5'-end with a radioactive isotope of phosphorus (5’-^33^P-labelled primer); lanes 2–12, products of PEX using: lane 2, 2’-deoxythymidine triphosphate (dTTP); lane 3, 2’-deoxyuridine triphosphate (dUTP); lane 4, 2’-deoxycytidine triphosphate (dCTP); lane 5, 2-pyridone-2’-deoxyriboside triphosphate (dPyrTP); lane 6, dPyr^4OH^TP; lane 7, dPyr^4Cl^TP; lane 8, dPyr^4Br^TP; lane 9, dPyr^5COOH^TP; lane 10, 4-thio-dUTP; lane 11, dU^5CN^TP; lane 12, dU^5COOH^TP; lane 13, *N*^4^-amino-dCTP.

**Figure 2:**
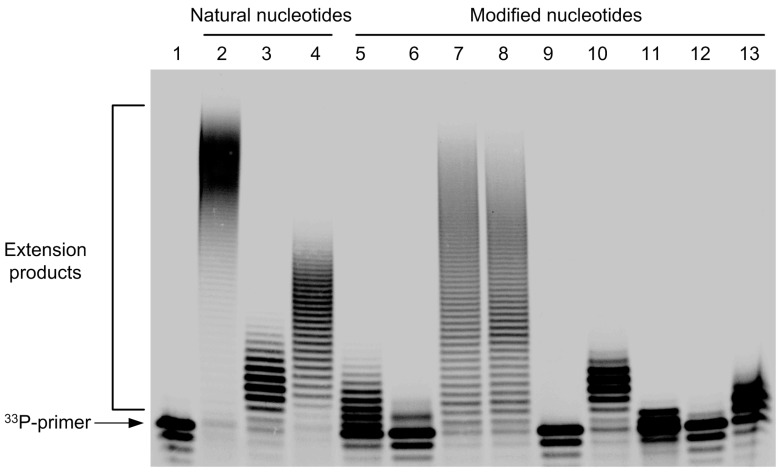
PAGE analysis of PEX with TdT in cacodylate/Co^2+^ buffer. Lane 1, 5′-^33^P-labelled primer; lanes 2–12, products of PEX using: lane 2, dTTP; lane 3, dUTP; lane 4, dCTP; lane 5, dPyrTP; lane 6, dPyr^4OH^TP; lane 7, dPyr^4Cl^TP; lane 8, dPyr^4Br^TP; lane 9, dPyr^5COOH^TP; lane 10, 4-thio-dUTP; lane 11, dU^5CN^TP; lane 12, dU^5COOH^TP; lane 13, *N*^4^-amino-dCTP.

Figure 1 and Figure 2 illustrate non-templated 3’-end elongation using 2-pyridone-based nucleoside triphosphates and compounds **3** (4-thio-2’-deoxyuridine triphosphate, 4-thio-dUTP), **4** (*N*^4^-amino-2’-deoxycytidine triphosphate, *N*^4^-amino-dCTP), **7** (5-cyano-2’-deoxyuridine triphosphate, dU^5CN^TP), **8** (5-carboxy-2’-deoxyuridine triphosphate, dU^5COOH^TP) catalyzed by TdT in Mg^2+^-containing and its optimal (Co^2+^-containing) buffer system, respectively. In general, the utilization of the optimal buffer for TdT resulted in more efficient incorporation of natural nucleotides as well as non-natural analogues (Figure 2). It should be noted that in the case of optimal reaction conditions TdT utilizes nearly every member of our library of non-natural nucleotides presented here whereas more than a half of compounds tested were unsuitable substrates for TdT under milder (glutamate/Mg^2+^) conditions. This in turn proves already established strict dependency of TdT catalytic activity and more importantly substrate specificity on reaction buffer.

It was previously demonstrated that 2-pyridone-based nucleotides are utilized by several template-dependent DNA polymerases [69]; however mostly inefficiently since only a single nucleotide was incorporated with no further elongation. Data provided in Figure 1 (lanes 7 and 8) revealed that longer products (up to ten extra nucleotides) were generated using 4-chloro-2-pyridone-2’-deoxyriboside triphosphate (dPyr^4Cl^TP) and 4-bromo-2-pyridone-2’-deoxyriboside triphosphate (dPyr^4Br^TP) as substrates than with equivalent concentrations of dUTP and dCTP suggesting that these compounds were better substrates for TdT. In addition, a substantial increase in the length of the elongation products by these two compounds in optimal environment was observed (Figure 2, lanes 7 and 8). Hence, in order to introduce varying number of monomers for the template-independent synthesis of halogenated nucleic acids, different buffers can be applied.

To the contrast, severe discrimination between two buffers was observed in the case of 2-pyridone-2’-deoxyriboside triphosphate (dPyrTP) and 4-hydroxy-2-pyridone-2’-deoxyriboside triphosphate (dPyr^4OH^TP) acting as substrates for TdT. Figure 1 illustrates that utilization of both dPyrTP and dPyr^4OH^TP by TdT was not detectable in glutamate/Mg^2+^ buffer (lanes 5 and 6). However in the presence of Co^2+^ TdT uses the former as substrate for the incorporation and subsequent elongation while only a single incorporation of dPyr^4OH^ is observed (Figure 2, lanes 5 and 6). Recently, it was demonstrated that binding affinity of TdT to the 3’-end of ON modified with 3-hydroxy-4-pyridone-based nucleotides depended on the Mg^2+^ concentration and caused formation of unfavourable secondary structure of 3’-terminus [70]. As a consequence after incorporation of single nucleotide further elongation was impeded. Our results support such interpretation and further suggest that not only buffer composition but also additional nucleobase modifications may lead to steric hindrance.

4-Thiouracil nucleobase is naturally occurring in some transfer RNA species and is widely used as a photoactivatable RNA-RNA, RNA-DNA, RNA-protein cross-linking agent [71]. Hence, T7 RNA polymerase uses 4-thio-UTP as a substrate during transcription in vitro, while it is known that 4-thiothymidine is readily incorporated into DNA during replication [45,46]. The data presented here revealed that 4-thio-dU was incorporated into ON with similar efficiency to its natural counterpart dU possibly due to its very similar structure. Despite the fact that Co^2+^-containing buffer system enhances utilization of certain nucleotide analogues, only a slight increase in incorporation of 4-thio-dU compared to glutamate/Mg^2+^ buffer was observed (Figure 2, lane 10). Nevertheless, these results suggest 4-thio-dUTP as a promising photo-affinity label for DNA.

*N*^4^-Amino-dCTP appeared to be a moderate substrate for TdT in both buffer systems compared to native nucleoside triphosphates. On the other hand, an ON bearing at least several hydrazine modifications could be further modified or cross-linked to nucleic acid molecule, protein or appropriately pre-treated surface.

Overall, dPyrTP, dPyr^4Cl^TP, dPyr^4Br^TP, 4-thio-dUTP and *N*^4^-amino-dCTP emerged as substrates used by TdT for incorporation and subsequent elongation whereas dPyr^4OH^ and dU^5CN^ were only incorporated but further elongation was halted. Only a single incorporation of dU^5CN^ is slightly confusing as it is known that TdT is able to incorporate modified pyrimidine nucleotides with bulky groups at fifth position [30,72]. Neither dPyr^5COOH^TP nor dU^5COOH^TP were used by TdT as substrates under both conditions tested. It might be speculated that a negative charge of both nucleotides was detrimental to an activity of TdT.

So far, no atoms have been unambiguously identified between the amino acid residues of TdT and the primer nucleobases that reside in sufficient proximity to one another to be considered a polar interaction, thus indicating that binding must rely entirely upon interaction with the sugar-phosphate backbone. It is assumed that the broad substrate specificity of TdT is based on polymerase insensitivity to the chemical structure of an incoming dNTP although the structure and accessibility of the 3’-terminus of ONs is crucial [26,28]. In addition, it has been proposed that kinetics of extension does not appear to be influenced by π-electron surface area of nucleotide analogues but rather by the size of modified nucleotides [11]. For example, it was shown that smaller nucleotides bearing 5-fluoroindolyl- and 5-nitroindolyl- modifications were more efficiently elongated whereas their bulkier counterparts (e.g., 5-napthylindolyl-modified), have been refractory to elongation. Hence, the inability to elongate large, bulky, non-natural nucleotides likely results from steric constraints [11,73]. However, the clear difference between efficiency of incorporation of dPyr^4OH^, dPyr^4Cl^ (or dPyr^4Br^) and 4-thio-dU as well as poor elongation of 5-cyano-modified dU by TdT cannot be explained in this way. TdT requires at least three deoxynucleotide residues on the primer strand for an efficient catalysis of the tailing reaction [31]. Subsequently, when polymerase reaches the extended section, 3’-end modifications could block the entrance of an incoming dNTP by enhancing primer affinity to TdT. Consequently reaction may stall explaining why only limited number of residues are appended, e.g., in the case of 4-thio-dU. The same result would also be observed if the 3’-modified primer fails to interact with the active center of TdT or it folds into unfavourable secondary structures as in the case of pyrene-2’-deoxynucleotide [26] or 2-methyl-3-hydroxypyridin-4(1*H*)-one 2’-deoxynucleotide (dH) [74]. In our hands TdT does not extend the 4-thio-dU-elongated primer in the presence of deoxythymidine triphosphate (dTTP) (data not shown); a similar effect has been observed with dH [70].

### 2.3. UV Cross-Linking of Oligonucleotides to Terminal Deoxynucleotidyl Transferase

In order to examine the potential of thio-modified ON to serve as a cross-linking agent, UVA-induced linking assay was conducted. Successful PEX using both 4-thio-dUTP and dUTP (a control reaction) were carried out where an average of 6 and 15 nucleotides were incorporated, respectively (data not shown). The next step was to select the model protein of interest to be cross-linked to 4-thiouridine-containing ON (4-thio-dU-ON). It was decided to cross-link the modified ON to TdT for several reasons. First of all, it is obvious that using TdT eliminates the need to search for proteins which specifically interact with the 3’-end of modified ONs. Secondly, it could be anticipated that, after catalysis, thio-groups would be located adjacent to amino acid side chains in the active site of TdT and thus revealing an actual interaction with nucleobase(s). Due to the fact that the generation of photo-induced complexes strongly depends on the distance between a photo-reactive group and amino acid residues it has been suggested that a successful cross-linking would be an evidence, even if indirect, of interactions between nucleobases and TdT.

**Figure 3:**
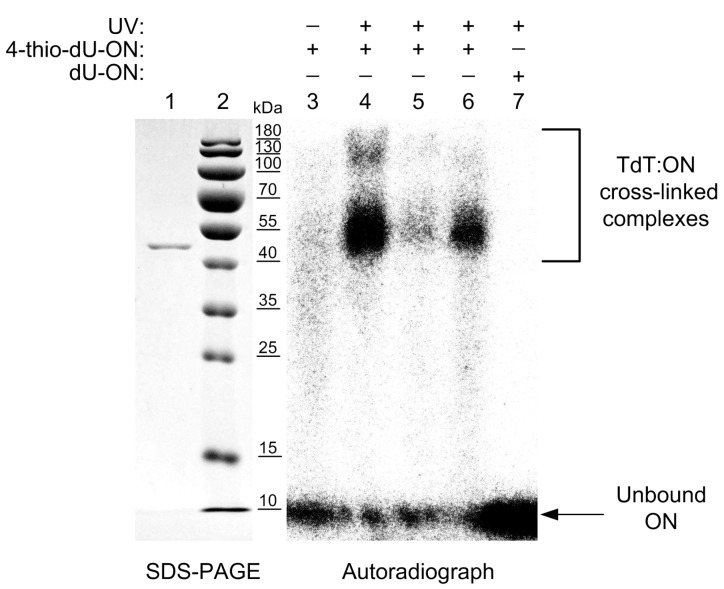
PAGE analysis of cross-linked complexes of 4-thio-dU-ONs and TdT. Lane 1, recombinant TdT; lane 2, molecular mass marker (kDa); lane 3, 4-thio-dU-ON:TdT UV-free control; lane 4, cross-linked complexes of 4-thio-dU-ON:TdT, dose of UV irradiation ~17.2 J/cm^2^; lane 5, cross-linked complexes of 4-thio-dU-ON:TdT, dose of UV irradiation ~4.6 J/cm^2^; lane 6, cross-linked complexes of 4-thio-dU-ON:TdT (excess of TdT), dose of UV irradiation ~17.2 J/cm^2^; lane 7; dU-ON:TdT control, dose of UV irradiation ~17.2 J/cm^2^.

Figure 3 shows the effect of exposing PEX mixtures to 365 nm UV light. Based on the slower mobility of the ^33^P label it was clear that the 4-thio-dU-ONs cross-linked to TdT (Figure 3, lanes 4–6). Data provided in Figure 3 (lanes 4 and 5) indicate that induction of cross-links depended upon a dose of UVA irradiation. A vast excess of TdT over ON did not improve cross-linking efficiency (Figure 3, lane 6). Moreover, it was elucidated that no cross-linked complexes were detected during irradiation at lower intensity (data not shown).

The free TdT has a molecular mass of 45–47 kDa as determined by sodium dodecyl sulfate-polyacrylamide gel electrophoresis (SDS–PAGE), whereas cross-linked complex run at the position of 55 kDa marker (Figure 3, lane 1 and lanes 4–6, respectively). This suggests a 1:1 covalent complex between TdT and 4-thio-dU-ON (molecular mass of 8–10 kDa). A second complex could be noted with a molecular mass of ~130 kDa though exact constitution of this heavier complex could not be ascertained easily (Figure 3, lane 4). It might be speculated that formation of more complicated cross-links was inevitable since ON could contain up to ten 4-thiouracil nucleobases, which theoretically might be triggered to generate at least several cross-links, whether it would be covalent bonds between two ONs, two protein molecules or aggregates of mixed origin. No cross-linking was observed in the absence of UV light and in the presence of dU-ONs.

TdT has a pivotal role in vivo to generate junctional diversity during V(D)J recombination by adding random nucleotides [20]. A detailed mechanism of antibody gene recombination still needs to be puzzled out. Although the biological role of TdT is tightly connected to its ability to utilize a wide variety of substrates in vitro and in vivo, there is no exact explanation on substrate selectivity of TdT up to date [75]. Data presented here slightly contradicts with the already established assumption that a broad substrate selectivity of TdT is based on unspecific interaction with sugar-phosphate backbone rather than specific contacts with nucleobases. Formation of UV-induced complexes between 4-thio-dU-ONs and TdT suggests an existing juxtaposition of the 4-thiouracil base with the appropriate amino acid side chains in the TdT.

All these observations clearly show that multiple interactions between the substrate and TdT take place and the nature of nucleobase plays an important role with respect to nucleotide selection and chain elongation. Therefore, further studies are needed to elucidate a substrate selectivity of TdT.

## 3. Materials and Methods

### 3.1. General Information

Chemicals and solvents were purchased from Sigma-Aldrich (Steinheim, Germany) and Alfa Aesar (Karlsruhe, Germany) and used without further purification. Thin-layer chromatography (TLC) was carried out on TLC aluminium sheets coated with silica gel 60 F254 (Merck, Darmstadt, Germany) and column chromatography on silica gel 60 (0.063–0.200 nm) (Merck). Reverse phase chromatography was carried out on Grace C-18 flash cartridges (BÜCHI, Flawil, Switzerland). Purification of nucleotides was carried out on diethylaminoethyl (DEAE) Sephadex A-25 columns (GE Healthcare, Helsinki, Finland) with a linear (0.05–0.4 M) gradient of LiCl as a mobile phase. Melting points were determined with a MEL-TEMP melting point apparatus (Electrothermal, Staffordshire, UK) in capillary tubes and are not corrected. NMR spectra were recorded in DMSO-*d_6_* or D_2_O on an Ascend 400: ^1^H-NMR–400 MHz, ^13^C-NMR–100 MHz and ^31^P-NMR–162 MHz (Bruker, Billerica, MA, USA). Chemical shifts (δ) are reported in ppm relative to the solvent resonance signal as an internal standard. UV spectra were recorded on a Lambda 25 UV/VIS spectrometer (Perkin Elmer, Singapore). High-performance liquid chromatography mass-spectrometry (HPLC-MS) analyses were performed using a high performance liquid chromatography system, equipped with a photo diode array detector (SPD-M20A) and a mass spectrometer (LCMS-2020, Shimadzu, Kyoto, Japan) equipped with an electrospray ionization (ESI) source. The chromatographic separation was conducted using a YMC Pack Pro column, 3 × 150 mm (YMC, Kyoto, Japan) at 40 °C and a mobile phase that consisted of 0.1% formic acid water solution or 5 mM ammonium acetate buffer (solvent A), and acetonitrile (solvent B). Mass spectrometry data was acquired in both positive and negative ionization mode and analyzed using the LabSolutions LCMS software (Software version 5.42 SP6, Shimadzu, Kyoto, Japan).

### 3.2. Synthesis of 4-Thio-2'-deoxyuridine (***2***)

(i) 3’,5’-Bis-*O*-acetyl-2’-deoxyuridine (**1i**). A mixture of acetic anhydride (804 μL, 8.52 mmol) and sodium acetate (116 mg, 1.42 mmol) was heated for 10 min at 90 °C. Then 2’-deoxyuridine (**1)** (648 mg, 2.84 mmol) was added to the hot solution and stirred for 30 min. After the reaction was completed (TLC), the mixture was quenched with sodium bicarbonate and extracted with chloroform. The organic phase was dried (Na_2_SO_4_) and evaporated under reduced pressure. The residue was purified by column chromatography (silica gel, chloroform/methanol mixture, 10:0→10:1). Yield 828 mg (93%), white solid, melting point (mp) 85–88 °C. retention factor (R_f_) = 0.75 (CHCl_3_/MeOH 5:1). MS (ESI^+^): *m/z* 313.00 [M+H]^+^, 311.00 [M-H]^−^. UV (CH_3_OH) λ_max_ (log ε) 262 (9.60) nm. ^1^H-NMR (DMSO-*d_6_*): δ = 2.06 (s, 3H, CH_3_), 2.07 (s, 3H, CH_3_), 2.28–2.34 (m, 1H, CH_2_), 2.43–2.46 (m, 1H, CH_2_), 4.16–4.19 (m, 1H, CH), 4.21–4.24 (m, 2H, CH_2_), 5.16–5.20 (m, 1H, CH), 5.71 (d, 1H, *J* = 8.1 Hz, CH=CH), 6.16 (dd, 1H, *J* = 6.2, 8.2 Hz, CH), 7.66 (d, 1H, *J* = 8.1 Hz, CH=CH), 11.40 (s, 1H, NH). ^13^C-NMR (DMSO-*d_6_*): δ = 21.05, 21.23, 36.18, 64.10, 74.38, 81.65, 84.91, 102.72, 140.83, 150.83, 163.44, 170.47, 170.61.

(ii) 3’,5’-Bis-*O*-acetyl-4-thio-2’-deoxyuridine (**1ii**). To a solution of 3’,5’-bis-*O*-acetyl-2’-deoxyuridine (818 mg, 2.62 mmol) in toluene (43 mL ) Lawesson’s reagent (636 mg, 1.57 mmol) was added. The reaction mixture was stirred for 2 h at 90 °C. After the reaction was completed (TLC), the mixture was quenched with sodium bicarbonate and extracted with chloroform. The organic phase was dried (Na_2_SO_4_) and evaporated under reduced pressure. The residue was purified by column chromatography (silica gel, chloroform/methanol mixture, 10:0→10:1). Yield 850 mg (98%), yellowish oil, R_f_ = 0.56 (CHCl_3_/MeOH 9:1). MS (ESI^+^): *m/z* 329.00 [M + H]^+^, 326.95 [M-H]^−^. UV (CH_3_OH) λ_max_ (log ε) 330 (16.50) nm. ^1^H-NMR (DMSO-*d_6_*): δ = 2.05 (s, 3H, CH_3_), 2.06 (s, 3H, CH_3_), 2.36–2.39 (m, 1H, CH_2_), 2.45–2.48 (m, 1H, CH_2_), 4.20–4.27 (m, 3H, CH, CH_2_), 5.17–5.22 (m, 1H, CH), 6.07–6.11 (m, 1H, CH), 6.37 (d, 1H, *J* = 7.6 Hz, CH=CH), 7.57 (d, 1H, *J* = 7.6 Hz, CH=CH), 12.78 (s, 1H, NH). ^13^C-NMR (DMSO-*d_6_*): δ = 21.04, 21.22, 36.59, 64.02, 74.30, 82.10, 85.91, 113.39, 136.16, 148.08, 170.47, 170.60, 190.75.

(iii) 4-Thio-2’-deoxyuridine (**2**). A mixture of 3’,5’-bis-*O*-acetyl- 4-thio-2’-deoxyuridine (840 mg, 2.55 mmol) and 1M sodium methylate (5.1 mL) was stirred for 30 min at room temperature. The reaction was monitored with TLC. The crude reaction mixture was purified by reverse phase column chromatography (C-18 cartridges, water/methanol mixture, 10:0→10:2). The solvents were removed under reduced pressure to afford yellowish solid reaction product, mp 147–150 °C. Yield 438 mg (70%), R_f_ = 0.19 (CHCl_3_/MeOH 9:1). MS (ESI^+^): *m/z* 242.95 [M − H]^−^. UV (H_2_O) λ_max_ (log ε) 332 (16.50) nm. ^1^H-NMR (DMSO-*d_6_*): δ = 2.03–2.06 (m, 1H, CH_2_), 2.11–2.15 (m, 1H, CH_2_), 3.56–3.61 (m, 2H, CH_2_), 3.79–3.84 (m, 1H, CH), 4.20–4.24 (m, 1H, CH), 4.93 (bs, 1H, OH), 5.25 (bs, 1H, OH), 6.09 (t, 1H, *J* = 6.6 Hz, CH), 6.25 (d, 1H, *J* = 7.4 Hz, CH=CH), 7.64 (d, 1H, *J* = 7.4 Hz, CH=CH), 11.79 (s, 1H, NH).^13^C-NMR (DMSO-*d_6_*): δ = 40.46, 49.05, 61.64, 70.70, 85.39, 88.00, 113.25, 135.39, 192.53. The NMR data were consistent with those reported previously [59].

### 3.3. Synthesis of 4-Thio-2'-deoxyuridine-5'-triphosphate (***3***)

To a suspension of synthesized 4-thio-2’-deoxyuridine (**2**, 125 mg, 0.51 mmol), tributylamine (244 µL, 1.02 mmol) in trimethyl phosphate (1.5 mL) cooled to 0 °C, phosphorous oxychloride (52 µL, 0.56 mmol) was added and the reaction mixture was stirred at 0 °C for 90 min. After the reaction was completed (TLC), tributylamine (87 µL, 0.37 mmol) and 0.5 M tributylammonium pyrophosphate solution ((NHBu_3_)_2_H_2_P_2_O_7_, 5.12 mL) in acetonitrile were added dropwise. After stirring for 15 min at 0 °C the reaction mixture was poured into ice-water and neutralized with saturated sodium bicarbonate solution. The reaction mixture was purified by ion exchange chromatography on DEAE-Sephadex A25 column (30 mL) with a linear gradient (0.05–0.4 M) of LiCl as the mobile phase. The product was eluted with 0.3 M LiCl, the solution was concentrated under reduced pressure to several mililiters and poured into a 40 mL mixture of acetone/methanol, 4:1. The formed precipitate was collected by centrifugation (4000 rpm, 10 min) and twice washed with a mixture of acetone/methanol, 4:1. The nucleotide was dissolved in 2 mL of water and evaporated under reduced pressure. Slightly acidic solution of nucleotide was neutralized with 1 M sodium hydroxide solution to pH 7.0. The synthesis and purification afforded 0.102 mmol (20% yield) of 4-thio-2’-dUTP. Compound was quantified by the extinction coefficient of 16.500 M^−1^ × cm^−1^ at 332 nm. R_f_ = 0.16 (dioxane/*i*PrOH/H_2_O/NH_4_OH 4:2:5:1). MS (ESI^+^): *m/z* 484.95 [M + H]^+^, 482.90 [M-H]^−^. UV (H_2_O) λ_max_ (log ε) 332 (16.50) nm. ^1^H-NMR (D_2_O): δ = 2.18–2.26 (m, 1H, CH_2_), 2.21–2.25 (m, 1H, CH_2_), 4.12–4.15 (m, 3H, CH_2_, CH), 4.50–4.54 (m, 1H, CH), 6.19 (t, 1H, *J* = 6.5 Hz, CH), 6.57 (d, 1H, *J* = 7.6 Hz, CH=CH), 7.75 (d, 1H, *J* = 7.6 Hz, CH=CH). ^31^P-NMR (D_2_O): δ = –20.39 (t, *J* = 18.5 Hz, Pβ); −10.85 (d, *J* = 18.6 Hz, Pα); –5.19 (d, *J* = 18.7 Hz, Pγ).

### 3.4. Synthesis of N^4^-Amino-2'-deoxycytidine-5'-triphosphate (***4***)

To a solution of 4-thio-2’-deoxyuridine triphosphate **3)**(32 mg, 0.066mmol) in water (2 mL) hydrazine hydrate (7 mg, 0.13 mmol) was added and the reaction mixture was stirred at room temperature for 18 h. After the reaction was completed (TLC), the reaction mixture was purified by ion exchange chromatography on DEAE-Sephadex A25 column (20 mL) with a linear gradient (0.05–0.4 M) of LiCl as the mobile phase. The product was eluted with 0.25–0.3 M LiCl, the solution was concentrated under reduced pressure to several mililiters and desalted using reverse phase column C-18. The synthesis and purification afforded 0.044 mmol (66% yield) of *N*^4^-amino-dCTP. Compound was quantified by the extinction coefficient of 13.700 M^−1^ × cm^−1^ at 274 nm. Rf = 0.12 (dioxane/*i*PrOH/H_2_O/NH_4_OH 4:2:5:1). MS (ESI^+^): *m/z* 483.00 [M+H]^+^, 480.95 [M − H]^−^. UV (H_2_O) λ_max_ (log ε) 274 (13.70) nm. ^1^H-NMR (D_2_O-): δ = 2.60–2.68 (m, 1H, CH_2_), 2.73–2.81 (m, 1H, CH_2_), 4.31–4.39 (m, 3H, CH_2_, CH), 4.43–4.41 (m, 1H, CH), 6.38 (t, 1H, *J* = 6.3 Hz, CH), 6.63 (d, 1H, *J* = 7.9 Hz, CH=CH), 8.09 (d, 1H, *J* = 7.9 Hz, CH=CH). ^31^P-NMR (D_2_O-): δ = −19.09 (t, *J* = 14.9 Hz, Pβ); −10.05 (d, *J* = 15.0 Hz, Pα); −5.86 (d, *J* = 15.0 Hz, Pγ). The NMR spectra were consistent with the data reported in [61].

### 3.5. Synthesis of 5-Cyano-2ʹ-deoxyuridine (***5***) and 5-Carboxy-2ʹ-deoxyuridine (***6***)

To a solution of 5-cyanouracil or 5-carboxyuracil (1.32 mmol) in dichloromethane (6 mL) hexamethyldisilazane (HMDS, 550 μL, 2.64 mmol) and trimethylsilyl chloride (TMSCl, 168 μL, 1.32 mmol) were added. The mixture was heated for 4 h at 80 °C. Then the mixture was cooled to room temperature and a solution of 1,3,5-*O*-triacetyl-2-deoxyribose (342 mg, 1.32 mmol) in dichloromethane (7 mL) and SnCl_4_ (309 μL, 2.64 mmol) were added. The reaction mixture was stirred for 5 h at room temperature. After the reaction was completed (TLC), the mixture was diluted with chloroform and washed with sodium carbonate solution. The organic phase was dried (Na_2_SO_4_) and evaporated under reduced pressure. The residue was purified by column chromatography (silica gel, chloroform/methanol mixture, 10:0→10:1). The acetylated nucleosides were de-protected with 1 M sodium methylate solution. The crude reaction mixtures were purified by reverse phase column chromatography (C-18 cartridges, water/methanol mixture, 10:0→10:2). The solvents were removed under reduced pressure to afford 5-substituted 2’-deoxyuridine derivatives **5** and **6**.

*5-Cyano-2’-deoxyuridine* (**5**). Yield 116 mg (35%), white solid, decomposes at 220 °C. R_f_ = 0.25 (CHCl_3_/MeOH 9:1). MS (ESI^+^): *m/z* 252.05 [M − H]^−^. UV (CH_3_OH) λ_max_ (log ε) 277 (11.70) nm. ^1^H-NMR (DMSO-*d_6_*): δ = 2.09–2.13 (m, 2H, CH_2_), 3.54–3.58 (m, 1H, CH_2_), 3.60–3.64 (m, 1H, CH), 3.86–3.90 (m, 1H, CH_2_), 4.23–4.27 (m, 1H, CH), 6.09 (t, 1H, *J* = 6.3 Hz, CH), 8.64 (s, 1H, C=CH). ^13^C- NMR (DMSO-*d_6_*): δ = 40.87, 61.97, 70.81, 88.19, 88.44, 90.73, 115.19, 146.71, 149.76, 160.51. The NMR spectra were in agreement with the data published in [62].

*5-Carboxy-2-deoxyuridine* (**6**). Yield 140 mg (40%), white solid, decomposes at 250 °C. R_f_ = 0.15 (CHCl_3_/MeOH 5:1). MS (ESI^+^): *m/z* 273.00 [M + H]^+^, 271.00 [M − H]^−^. UV (H_2_O) λ_max_ (log ε) 275 (9.70) nm. ^1^H-NMR (DMSO-*d_6_*): δ = 2.28–2.35 (m, 2H, CH_2_), 3.78–3.82 (m, 2H, CH_2_), 4.19–4.23 (m, 1H, CH), 4.55–4.59 (m, 1H, CH), 6.29 (t, 1H, *J* = 6.4 Hz, CH), 8.84 (s, 1H, C=CH). ^13^C-NMR (DMSO-*d_6_*): δ = 39.17, 61.92, 70.81, 86.34, 87.73, 105.17, 148.71, 158.76, 162.53, 167.14. The ^1^H-NMR spectrum was consistent with the previously reported one [64].

### 3.6. Synthesis of 5-Substituted Deoxyuridine TriphosphateDerivatives ***7*** and ***8***

Nucleoside (**5** or **6**, 0.2 mmol) was dissolved in trimethylphosphate (1 mL) and cooled to 0 °C. Tributylamine (71 µL 0.3 mmol) was added, followed by phosphorous oxychloride (30 µL, 0.3 mmol) 5 min later. After several hours of stirring at 0–4 °C (TLC), tributylamine (42 mL, 0.2 mmol) and 0.5 M (NHBu_3_)_2_H_2_P_2_O_7_ in acetonitrile (2 mL) were added dropwise. After stirring for 10–15 min the reaction mixture was poured into water and neutralized with saturated sodium bicarbonate solution. The crude reaction mixture was purified by ion exchange chromatography on DEAE-Sephadex A25 column (20 mL) with a linear gradient (0.05–0.4 M) of LiCl as mobile phase. The product was eluted with 0.25–0.3 M LiCl, the solution was concentrated under reduced pressure to several mililiters and poured into a 40 mL mixture of 4:1 acetone/methanol,. The formed precipitate was collected by centrifugation (4000 rpm, 10 min) and washed twice with a mixture of 4:1 acetone/methanol. The nucleotide was dissolved in water (2 mL) and evaporated under reduced pressure. Slightly acidic solution of nucleotide was neutralized with 1 M NaOH solution to pH 7.0.

*5-Cyano-2’-deoxyuridine-5’-triphosphate* (**7**). The synthesis and purification afforded 0.039 mmol (19.5% yield) of 5-cyano-2’-dUTP. Compound was quantified by the extinction coefficient of 11.700 M^−1^ × cm^−1^ at 277 nm. R_f_ = 0.16 (dioxane/*i*PrOH/H_2_O/NH_4_OH 4:2:5:1). MS (ESI^+^): *m/z* 491.90 [M − H]^−^. UV (H_2_O) λ_max_ (log ε) 277 (11.70) nm. ^1^H-NMR (D_2_O): δ = 2.30–2.35 (m, 1H, CH_2_), 2.65–2.72 (m, 1H, CH_2_), 4.01–4.04 (m, 2H, CH_2_), 4.16–4.20 (m, 1H, CH), 4.52–4.56 (m, 1H, CH), 6.12 (m, 1H, CH), 8.52 (s, 1H, CH=C). ^31^P-NMR (D_2_O): δ = −20.69 (t, *J* = 19.1 Hz, Pβ), –10.95 (d, *J* = 18.7 Hz, Pα), −5.33 (d, *J* = 19.1 Hz, Pγ).

5*-Carboxy-2’-deoxyuridine-5’-triphosphate* (**8**). The synthesis and purification afforded 0.041 mmol (20.5% yield) of 5-carboxy-2’-dUTP. Compound was quantified by the extinction coefficient of 9.700 M^−1^ × cm^−1^ at 275 nm. R_f_ = 0.10 (dioxane/*i*PrOH/H_2_O/NH_4_OH 4:2:5:1). MS (ESI^+^): *m/z* 510.85 [M − H]^−^. UV (H_2_O) λ_max_ (log ε) 275 (9.70) nm. ^1^H-NMR (D_2_O, 400 MHz): δ = 2.31–2.37 (m, 1H, CH_2_), 2.65–2.72 (m, 1H, CH_2_), 4.01–4.04 (m, 2H, CH_2_), 4.10–4.14 (m, 1H, CH), 4.49–4.53 (m, 1H, CH), 6.16 (m, 1H, CH), 8.43 (s, 1H, CH=C). ^31^P-NMR (D_2_O, 162 MHz): δ = −20.58 (t, *J* = 17.9 Hz, Pβ), −10.91 (d, *J* = 18.3 Hz, Pα), −5.28 (d, *J* = 17.9 Hz, Pγ). The NMR spectra were consistent with those reported previously [76].

### 3.7. Synthesis of 2-Pyridone Nucleoside Triphosphates

(3-Hydroxy-5-(2-oxo-1-pyridyl)tetrahydrofuran-2-yl)methyl triphosphate (dPyrTP), (3-hydroxy-5-(4-hydroxy-2-oxo-1-pyridyl)tetrahydrofuran-2-yl)methyl triphosphate (dPyr^4OH^TP), (5-(4-chloro-2-oxo-1-pyridyl)-3-hydroxytetrahydrofuran-2-yl)methyl triphosphate (dPyr^4Cl^TP), (5-(4-bromo-2-oxo-1-pyridyl)-3-hydroxytetrahydrofuran-2-yl)methyl triphosphate (dPyr^4Br^TP), and (5-(5-carboxy-2-oxo-1-pyridyl)-3-hydroxytetrahydrofuran-2-yl)methyl triphosphate (dPyr^5COOH^TP) were prepared as described previously [70].

### 3.8. Incorporation of Modified Nucleotides by Terminal Deoxynucleotidyl Transferase

The DNA primer (5’-TAATACGACTCACTATAGGGAGA-3’) was purchased from Metabion (Planegg/Steinkirchen, Germany), TdT was purchased from Thermo Fisher Scientific (Vilnius, Lithuania). The primer sequence was 5’-^33^P labelled by treatment with [γ-^33^P]-ATP (~3000 Ci mmol^−1^, Perkin Elmer) using T4 polynucleotide kinase (Thermo Fisher Scientific) according to manufacturer’s recommendations. The 5’-labelled primer was desalted using Zeba^TM^ Spin desalting columns (7K MWCO, Thermo Fisher Scientific). The reaction mixtures were prepared in a total volume of 10 µL by adding 5’-^33^P-labelled primer (0.5 µL, 5 pmol), 2 µL 5 × TdT reaction buffer (1× buffer contains 200 mM potassium cacodylate, 25 mM Tris, 0.01% Triton X-100, 1 mM CoCl_2_, pH 7.2, included in TdT kit) or 5 × glutamate buffer (1 × buffer contains 20 mM sodium glutamate, 20 mM NaCl, 10 mM DTT, 0.5% Triton X-100, 1 mM MgCl_2_, pH 8.2), modified dNTP (final concentration 10 µM), TdT (0.5 µL, 50 nM) and distilled water. The reaction mixtures were incubated at 37 °C for 5 min. The reactions were quenched with 20 µL of loading solution (95% *v/v* formamide, 20 mM EDTA, 0.03% *w/v* bromophenol blue, 0.03% *w/v* xylene cyanol). Reaction products were analyzed by denaturing PAGE (15% polyacrylamide, 8 M urea) in TBE (Tris/Borate/Ethylendiaminetetraacetic acid, EDTA) buffer (89 mM Tris, 89 mM boric acid, 2 mM EDTA, pH 8.3) and visualized using a FLA-5100 imaging system (FUJIFILM, Tokyo, Japan).

### 3.9. Formation of 4-Thio-dU-ON:Protein Cross-links

The cross-linking apparatus was constructed with slight modifications as described previously [77]. It consisted of an ice container, 96-well plate, a sheet of parafilm and a UV light source (Epileds, Tainan, Taiwan). Samples were irradiated at 365 ± 5 nm (200–220 mW/cm^2^) 5 and 15 mm away from the surface of the light source, which provided dose of UV irradiation ~17.2 J/cm^2^ and ~4.6 J/cm^2^, respectively. A sheet of parafilm was placed over the top of 96-well plate, and taped to the plate on all four sides. Each well was pressed to create a shallow groove. The plate was kept on ice before and during irradiation.

UV cross-linking of 4-thio-dU-elongated DNA primer (5’-^33^P-labelled) to TdT was carried out after PEX. PEX were performed in Co^2+^-containing buffer as described above. Immediately after incubation, the reaction mixtures were chilled on ice and transferred as 10 µL drops to the wells on the parafilm tape. The ice container was placed underneath a 365 nm UV, so that the samples were 5 and 15 mm from the surface of the light source. Following incubation on ice the samples were irradiated at 365 nm for 5 min. Then the samples were transferred from parafilm wells to microtubes and quenched with 20 µL of loading solution. To verify the products of elongation reactions using 4-thio-dUTP and dUTP by TdT samples were examined on 15% polyacrylamide gel containing 8 M urea. Alternatively, to examine potential TdT-ON cross-links generated by irradiation, the samples were supplemented with SDS loading dye, heated for 5 min at 95 °C, and analyzed by electrophoresis on a 14% *w/v* SDS-PAGE gel. Proteins were stained with Coomasie Briliant Blue staining solution (Applichem, Darmstadt, Germany). TdT and radiolabelled-ONs cross-links were visualized using an FLA-5100 imaging system (FUJIFILM).

## 4. Conclusions

We have synthesized novel modified nucleotides that can be incorporated into DNA sequences by terminal deoxynucleotidyl transferase in the primer-extension reaction. We have succeeded in the enzymatic elongation by 4-thio-2’-deoxyuridine, *N*^4^-amino-2’-deoxycytidine and 2-pyridone base-bearing nucleotides utilizing template-independent TdT, which produced the artificial DNA fragments tailed with several to tens nucleotides at the 3′-end. For example, 4-thio-dU-label has potential in studying specific proteins which interact exclusively with the DNA 3’-end, hydrazine-bearing ONs could be successfully applied in a conjugation to improve in vivo delivery of therapeutic ONs or immobilization, whereas halogenated-ONs seem to be irreplaceable radiosensitizing agents. Enzymatic post-synthetic modification of ONs with various photo- and chemically-reactive groups is in progress towards further use for the modification of DNA or RNA. Thus, this study would open many possibilities for future applications, especially in the development of photo-labels, sensors, and self-assembling structures.
